# Effects of mesh-related complications in vaginal surgery on quality of life

**DOI:** 10.1007/s00192-018-3680-9

**Published:** 2018-06-16

**Authors:** Claudia R. Kowalik, Mariëlle M. E. Lakeman, Alyde T. de Kraker, Jan Paul W. R. Roovers

**Affiliations:** 1 Department of Obstetrics and Gynecology, Academic Medical Center, Room H4-272, PO Box 22660, 1100 DD Amsterdam, The Netherlands; 2 Department of Obstetrics and Gynecology, BovenIJ ziekenhuis, Statenjachtstraat 1, Po box 37610, 1030 BD Amsterdam, The Netherlands; 3 Department of Obstetrics and Gynecology, Academic Medical Center, Room H4-240-1, PO Box 22660, 1100 DD Amsterdam, The Netherlands

**Keywords:** Complications, Health-related quality of life, Prolapse, Vaginal mesh

## Abstract

**Introduction and hypothesis:**

Vaginal mesh surgery is subject of debate due to the impact of mesh-related complications on patient’s lives. Not all of these complications are symptomatic. Restoration of the anatomy and improvement of pelvic floor function as a result may counter the experienced discomfort related to adverse events. We hypothesized that health-related quality of life (HR-QoL) is comparable in women after vaginal mesh surgery regardless of the presence or absence of a mesh-specific complication.

**Methods:**

This was a cross-sectional study of 128 women who had vaginal mesh surgery in a Dutch university hospital between 2007 and 2012. HR-QoL was measured in women with and without mesh complications using standardized QoL questionnaires Urogenital Distress Inventory-6 (UDI-6), Incontinence Impact Questionnaire (IIQ), Defecation Distress Inventory (DDI), and Pelvic Organ Prolapse/Urinary Incontinence Sexual Function Questionnaire (PISQ-12). Complications were scored according to the International Urogynecological Association (IUGA) complication classification. Comparisons between groups were performed with Student’s* t* test and analysis of variance (ANOVA) test.

**Results:**

In 29 (23%) women, a mesh-related complication occurred. The domain scores of the UDI-6, DDI, IIQ, and PISQ showed no statistically significant differences between women with and without a mesh-related complication. A post hoc analysis showed similar HR-QoL for those in whom the complication had been resolved and those with persistent symptoms of the complication.

**Conclusion:**

Mesh surgery imposes specific complications. When counseling patients about the potential adverse events related to vaginal mesh surgery, it is important to inform them that mesh-related complications do not negatively affect QoL related to micturition, defecation, and sexual functioning.

## Introduction

The treatment of pelvic organ prolapse (POP) is challenging, since the recurrence rate after surgical treatment is high. It has been shown that 29% of women who undergo an operation for POP and/or urinary incontinence (UI) will have a reoperation [1]. In women with POP recurrence, a reoperation rate of 11.5% has recently been reported in a large Danish cohort study after 20 years of follow-up [2]. Polypropylene mesh was introduced as the possible solution to this high recurrence risk. The mesh induces a foreign body response and as part of that, new collagen and elastin are formed, consequently making the repair less dependent on the patient’s own connective tissue [3]. Altman and co-workers have shown that both objective and subjective cures are better following vaginal mesh surgery compared with native tissue repair. They reported a reintervention rate for mesh-related complications of 3.2%, but a recent multicenter randomized controlled trial (RCT) performed in the UK reported a reoperation risk for mesh-related complications in 9% of the patients [4, 5]. The risk on mesh-related complications was one of the main reasons for the US Food and Drug Administration (FDA) to issue public health notifications in 2008 and 2011 advising the restricted use of mesh and optimize patient counseling regarding the possible adverse events of mesh surgery [6].

Interestingly enough, the specific effects of mesh-related complications on the patient’s QoL have not yet been studied. Not all complications are symptomatic, and the advantages of restored anatomy and associated improved pelvic floor function may outweigh the discomfort related to surgery-related adverse events. To optimize patient counseling about mesh-specific adverse events, as recommended by the FDA and professional organizations, information about the effects of such complications on QoL is highly relevant. We hypothesized that HR-QoL is comparable in women after vaginal mesh surgery regardless of the presence or absence of a mesh-specific complication.

## Materials and methods

We performed a cross-sectional study in the Academic Medical Center (AMC) in Amsterdam, The Netherlands. The medical ethics committee of the AMC judged that the Medical Research Involving Human Subjects Act does not apply to this study, since the study only involved completing questionnaires and one additional pelvic floor examination that can be justified by the fact that mesh-related complications, if present, can be managed.

### Population

Women who had a vaginal polypropylene mesh procedure at the AMC between 2007 and 2012 were contacted by letter and asked for consent to participate in this study. Within 2 weeks after the letter had been sent, women were contacted by phone and asked if they were willing to participate in the study. Women who did not want to be contacted could opt out by sending an email. Indications to perform vaginal mesh surgery were recurrence of POP, women with a posterior vaginal wall prolapse after previous vaginal hysterectomy, or women participating in studies to evaluate the outcomes of vaginal mesh surgery [7–9].

### Procedures

Mesh kits implanted during the study period were Perigee™, Apogee™, Elevate® Anterior, and Elevate® Posterior (Astora Women’s Health, Eden Prairie, MN, US). The procedures were performed under general or spinal analgesia. The procedures were performed as indicated by the manufacturer of the mesh implant.

### Outcome measurements

We measured HR-QoL in women following mesh surgery defined as QoL related to micturition, defecation, and sexual functioning. This was assessed using the Dutch versions of the following validated HR-QoL questionnaires: Urogenital Distress Inventory-6 (UDI-6), Incontinence Impact Questionnaire (IIQ), Defecation Distress Inventory (DDI), Pelvic Organ Prolapse/Urinary Incontinence Sexual Function Questionnaire-12 (PISQ-12). Women were asked to fill out the questionnaires prior to their clinical examination.

1. The UDI-6 assesses the presence and experienced bother of pelvic floor symptoms associated with lower urinary tract dysfunction. Scores are divided into three domains: irritative, stress, and obstructive/discomfort symptoms. Scores range from 0 to 100 per domain, with 0 identifying patients who experience no bother related to micturition symptoms and 100 identifying patients who experience maximal bother [10, 11].
2. The IIQ measures the impact of UI on different aspects of QoL. The questions are divided into four domains: travel, physical activity, social relationships, and emotional health. Subscale scores range from 0 to 100. The total score is given by the sum of all subscale scores, ranging from 0 to 400. A higher score implicates more bother [12].
3. The DDI assesses the presence and experienced bother of defecatory symptoms. The questions are divided into four distinct domains: constipation, painful defecation, fecal incontinence, and flatus incontinence [13]. Each domain score ranges from 0 to 100, with 0 identifying patients without any defecatory symptoms and 100 identifying patients who encounter all possible symptoms and experience these as maximally bothersome [14].
4. The PISQ-12 assesses sexual functioning in women with POP and/or UI and addresses physical, behavioral–emotive, and partner-related aspects of sexual functioning. The sum score ranges from 0 to 48, with a higher score indicating better sexual functioning [15].

To assess the presence or absence of a mesh-related complication, participators were invited for a study visit during which their history was taken and a pelvic examination was performed by a gynecologist. The investigator was not blinded to the previously performed mesh procedure, and in some cases, the evaluator participated in the index surgery. During this visit, current symptoms of pelvic floor dysfunction or mesh-related complications such as pain or exposure, reinterventions for pelvic floor dysfunction, or complications related to surgery and adjuvant treatment (medication, surgery, or physiotherapy) were discussed. Complications were scored according to the IUGA classification of complications related directly to the insertion of prostheses or grafts in urogynecological surgery [16].

### Statistical analysis

Patient demographic and baseline characteristics were summarized using standard descriptive methods. HR-QoL was calculated by analyzing the outcome of the validated QoL questionnaires. Comparisons of the UDI-6, IIQ, DDI, and PISQ-12 between women with and without a mesh complication were performed using the independent samples* t* test. Comparison between more than two groups were performed with the analysis of variance (ANOVA) test.

Post hoc analyses were performed to evaluate if there were any statistically significant differences between women in which the complication was still or had been resolved. Data was analyzed using IBM SPSS Statistics 23.

## Results

The response rate was 68% (128/188 patients). Of these women, ten refused to be contacted by mail, three were deceased, 14 could not be reached, and 21 refused participation after they had been contacted by phone. Of the 140 women who consented to participate, 128 actually filled out the questionnaires. Most women did not give a specific reason for not being willing to participate. Baseline characteristics of respondents are shown in Table 1. Women who filled out the questionnaire and visited the clinic were significantly younger at the time of surgery than women who did not want to participate (*p* = 0.04). Most women (78%) had a mesh procedure because of recurrence. Almost all women who underwent primary mesh surgery participated in a clinical study. Ten women had had a previous mesh implantation for POP.

**Table 1:** Baseline characteristics of the study population

	No complication	Complication	*P* value
	*n* = 99	*n* = 29	
Patient demographics			
Age (years), mean (SD)	60 (11)	57 (9.3)	0.17
BMI, mean (SD)	26 (3.6)	27 (3.8)	0.09
Parity mean (SD)	2.3 (0.8)	2.6 (1.3)	0.28
Follow-up (months), median (range)	39 (17.5)	46 (19.5)	0.12
Surgical history			
Hysterectomy vaginal	41 (41%)	13 (45%)	0.27
Hysterectomy laparoscopic	0	1 (3%)	
Hysterectomy abdominal	11 (11%)	2 (7%)	
Type of mesh			
Perigee	19 (19%)	8 (28%)	0.02
Apogee	0	2 (7%)	
Elevate anterior	44 (44%)	9 (31%)	
Elevate posterior	35 (35%)	8 (28%)	
Mesh in multiple compartments	1 (1%)	2 (7%)	

Data are expressed as mean (SD), median (range), or absolute numbers (%)

*SD* standard deviation, BMI body mass index

Significance cutoff *p* < 0.05

From the respondents, 29 women (23%) had a complication during follow-up, 17 had an exposure, and 12 had long-lasting pain without exposure. The IUGA classification of these complications is depicted in Table 2.

**Table 2:** Frequency of the international Urogynecology Association/International Continence Society scoring system for mesh-related complications

Category	Total	Code	Frequency	Description
Category 1	12	1BcT2S8	1	Symptomatic, pain during sexual intercourse
		1BcT3S1	1	Symptomatic, pain during sexual intercourse
		1BcT4S2	1	Symptomatic, pain during sexual intercourse
		1BcT8S8	1	Symptomatic, pain during sexual intercourse
		1BdT3S4	1	Symptomatic, pain during physical activities
		1BeT1S3	1	Symptomatic, spontaneous pain
		1BeT2S3	1	Symptomatic, spontaneous pain
		1BeT3S4	1	Symptomatic, spontaneous pain
		1BeT4S2	1	Symptomatic, spontaneous pain
		1BeT4S3	1	Symptomatic, spontaneous pain
		1BeT4S5	1	Symptomatic, spontaneous pain
		1BeT8S5		Symptomatic, spontaneous pain
Category 2	10	2AaT2S1	1	Asymptomatic mesh exposure <1 cm
		2AaT4S2	3	Asymptomatic mesh exposure <1 cm
		2AaT4S8	1	Asymptomatic mesh exposure <1 cm
		2B8T4S1	1	Symptomatic mesh exposure <1 cm
		2BaT4S1	1	Symptomatic mesh exposure <1 cm, no pain
		2BaT4S2	1	Symptomatic mesh exposure <1 cm, no pain
		2BbT4S1	1	Symptomatic mesh exposure <1 cm, provoked pain
		2BbT4S2	1	Symptomatic mesh exposure <1 cm, provoked pain
Category 3	5	3AaT4S2	3	Asymptomatic mesh exposure >1 cm
		3BaT2S1	1	Symptomatic mesh exposure>1 cm, no pain
		3BaT4S1	1	Symptomatic mesh exposure>1 cm, no pain
	2	8AaT2S1	1	Asymptomatic mesh exposure, exposure size unknown
		8BaT2S1	1	Symptomatic mesh exposure, exposure size unknown

Functional outcome of women with and without a complication during follow-up is compared in Table 3. UDI, DDI, and IIQ scores did not significantly differ between groups. Post hoc analyses showed no statistically significant differences between women in whom the complication was still present or had been resolved.

**Table 3:** Health-related quality of life

	No complication	With complication	*P* value
	*n* = 99	*n* = 29	
UDI-6 mean (SD)			
Irritative subscale	31.1 ± 28.6	36.1 ± 31.7	0.43
Stress subscale	26.7 ± 26.6	28.8 ± 23.8	0.71
Obstructive subscale	23.6 ± 27.1	29.6 ± 32.1	0.33
IIQ mean (SD)			
Physical activity	23.3 ± 23.3	25.5 ± 27.2	0.68
Mobility	26.1 ± 24.6	22.4 ± 24.6	0.49
Social functioning	14.1 ± 18.8	15.6 ± 19.1	0.71
Emotional health	18.8 ± 20.2	19.3 ± 23.6	0.92
DDI			
Constipation	13.4 ± 20.6	9.0 ± 15.8	0.31
Painful defecation	9.4 ± 21.6	14.3 ± 29.3	0.34
Fecal incontinence	15.0 ± 22.4	13.7 ± 21.3	0.78
Flatus incontinence	27.3 ± 29.3	27.2 ± 30.7	0.98
PISQ-12 summary score	35.1 ± 6.7	36 ± 4.9	0.61

Not all women answered all questions

*SD* standard deviation, *UDI-6* Urogenital Distress Inventory, *IIQ* Incontinence Impact Questionnaire, *DDI* Defecatory Distress Inventory, *PISQ-12* Pelvic Organ Prolapse/Urinary Incontinence Sexual Function Questionnaire

Significance cutoff at *p* < 0.05

From respondents, 72 (56%) women reported being sexually active; they were significantly younger [54.5 years (SD 8.9) vs 64.8 years (SD 9.4); *p* = 0.00). In women with a mesh-related complication, 62% (18/29) were sexually active vs 55% in the group of women without a complication. Of women in whom the complication was still present during the follow-up visit, 63% was sexually active.

Whether refraining from sexual activity was caused by mesh complications is not known. Table 4 depicts the replies of sexually active women with and without complications. In the whole group of sexually active women, 69% was very or reasonably satisfied with their sex life (50/72). No statistically significant differences were found between total PISQ scores in women with [mean 35.1 (SD 6.7)] and without [mean 36 (SD 4.9)] mesh complications (*p* 0.61). Of the 29 patients with a complication, the complication resolved in 12. A post hoc analysis showed no statistical difference in total PISQ scores in women with a persistent complication or in whom the complication had been resolved.

**Table 4:** Sexual functioning

		No complication (*n* = 54)	With complications (*n* = 18)	*P* value (chi-square)
Satisfaction	Very/reasonably satisfied	36 (67%)	14 (78%)	0.54
	Not satisfied/not unsatisfied	10 (19%)	1 (6%)	
	Fairly/very dissatisfied	5 (9%)	3 (17%)	
Urinary incontinence during intercourse	Never	43 (80%)	14 (78%)	0.48
	Rarely/sometimes	7 (13%)	2 (11%)	
	Usually/always	2 (4%)	2 (11%)	
Fear of incontinence during intercourse	Never	31 (57%)	13 (72%)	0.77
	Rarely/sometimes	15 (28%)	4 (22%)	
	Usually/always	7 (13%)	1 (6%)	
Avoidance	Never	36 (67%)	14 (78%)	0.20
	Rarely/sometimes	12 (22%)	3 (17%)	
	Usually/always	4 (7%)	0	
Negative feelings	Never	28 (52%)	10 (56%)	0.67
	Rarely/sometimes	19 (35%)	7 (39%)	
	Usually/always	5 (9%)	1 (6%)	
Aroused	Never	1 (2%)	0	0.43
	Rarely/sometimes	12 (23%)	7 (39%)	
	Usually/always	39 (75%)	11 (61%)	
Orgasm	Never	7 (14%)	2 (11%)	0.45
	Rarely/sometimes	18 (35%)	3 (17%)	
	Usually/always	27 (52%)	13 (72%)	
Desire	Never	2 (4%)	0	0.37
	Less than once	7 (13%)	2 (11%)	
	Monthly	17 (32%)	10 (56%)	
	Weekly/daily	28 (52%)	6 (33%)	
Pain	Never	25 (49%)	7 (41%)	0.65
	Rarely/sometimes	15 (29%)	4 (24%)	
	Usually/always	11 (22%)	6 (35%)	
Erectile function problems	Never	30 (57%)	13 (72%)	0.44
	Rarely/sometimes	22 (42%)	5 (28%)	
	Usually/always	1 (2%)	0	
Orgasm intensity	Much more intense/more intense	6 (11%)	2 (11%)	0.51
	Same	25 (46%)	9 (50%)	
	Less/much less intense	18 (33%)	6 (33%)	

Not all women answered all questions

## Discussion

This study shows that disease-specific HR-QoL was comparable between women with and without mesh-related complications. Overall domain scores were in the lower range for the UDI-6, IIQ, and DDI, indicating less bother, and in the higher range for the PISQ-12, indicating better sexual functioning. Women with mesh-related complications were more often sexually active as those without, although this difference was not statistically significant.

Apart from our study, only one publication specifically evaluated the relationship between disease-related QoL and the occurrence of mesh-related complications. In the observational study among 114 patients undergoing transvaginal repair with mesh, no significant impact of mesh exposure on the patient’s QoL was observed, as with our observation. In addition, mean QoL scores were improved after mesh surgery in both the exposure and nonexposure group [17]. A possible explanation for the similar QoL between women with and without a mesh complication may be that micturition, defecation, and sexual function are mostly related to vaginal anatomy, which is optimized after surgery. The positive impact of restoration of anatomy and elimination of bothersome micturition and defecation symptoms after vaginal mesh surgery counters the negative impact of mesh-related complications.

No statistically significant differences were found in total PISQ scores between women with and without a complication. This is in contrast to the study by Milani et al., who performed an RCT comparing sexual function in women with recurrent POP having either native tissue repair or trocar-guided vaginal mesh surgery. They found that the presence of mesh exposure was independently associated with deterioration of PISQ scores [18]. The probable reason for not observing worsening sexual functioning in women with mesh-related complications is that we used implants with a lower density than Prolift, which was used in the study by Milani et al. [18]. It has been shown that lower mesh density is associated with less fibrosis and contraction compared with higher-density mesh [19].

The sexually active women in our study were relatively satisfied, with the interesting observation that women with a mesh complication, although not statistically significant, appeared to be more satisfied with their sex life. A possible explanation might be that after recovery of a complication, the mere fact of being able to have sexual intercourse again or the absence of pain complaints might be a big relief. Women with a mesh complication were more frequent sexually active. An explanation may be that more sexual activity increases the risk of exposure, as friction is a risk factor for exposure. Future research is needed to investigate whether this observation is a real phenomenon or related to low numbers. 

Some design-related issues in this study need to be addressed: Even though health domain scores were in the low range, they were slightly higher in our study compared with other studies that report on QoL in women having a vaginal mesh implant [4, 20]. This might be explained by the difference in duration of follow-up. We report domain scores after a median of 40 months, in contrast with the 12-month follow-up in those studies [4, 20]. We describe vaginal mesh procedures with implants that are no longer marketed. The Elevate® system implanted in most patients in our study was a single-incision technique that provided anterior or posterior repair as well as apical suspension by attachment of the mesh to the sacrospinous ligaments.

We believe results of this study can be extrapolated to other vaginal meshes that are at the market today. Currently available meshes often have similar insertion techniques and are also made of polypropylene, with a similar or even lighter mesh density. This study could be affected by selection bias. Women who consented to participate were of significantly younger age, and younger women may theoretically have better health and therefore may be prone to report a better subjective QoL. Only 58% of our study population reported to be sexually active. We do not know whether refraining from sexual activity was due to a mesh complication; however, this percentage of sexually active women is comparable with previous studies among women with mesh repair and conventional surgery [4, 20]. Comparison of PISQ scores between women with and without a complication showed no difference; however, groups were small.

Several points strengthen our study findings: First, the median follow-up was >3 years, and thus our study represents long-term data. Second, we used disease-specific validated HR-QoL questionnaires to assess subjective outcome measures. Disease-specific questionnaires provide higher face validity and more in-depth assessment of specific issues and concerns to the population under study compared with generic questionnaires, which reliably assess effects of treatment on HR-QoL [12]. Third, this report is one of few that specifically quantify the effect on sexual functioning in women with mesh-related complications.

In conclusion, this study shows that HR-QoL regarding micturition, defecation, and sexual function is comparable between women with and without a mesh-related complication after vaginal mesh surgery. This is important information in light of the ongoing debate regarding vaginal mesh surgery. The risk of (irreversible) effects of mesh-related complications is the reason many physicians and their patients refrain from vaginal mesh surgery. However, some patients, like women with prolapse recurrence, may benefit from vaginal mesh surgery. Based on this study, women could be informed that there is a risk of complications related to vaginal mesh surgery but that improved QoL related to pelvic floor function is likely to counteract the impact of the mesh-specific complication. This information facilitates a well-balanced treatment selection.
